# Supplementary material for: Soybean Mosaic Virus 6K1 Interactors Screening and GmPR4 and GmBI1 Function Characterization
Source: Int J Mol Sci. 2023 Mar 10;24(6):5304. doi: 10.3390/ijms24065304 (PMC10049162; doi:10.3390/ijms24065304)
Supplement: Supplementary file 1 [file ijms-24-05304-s001.zip › ijms-2172060-supplementary.pdf]

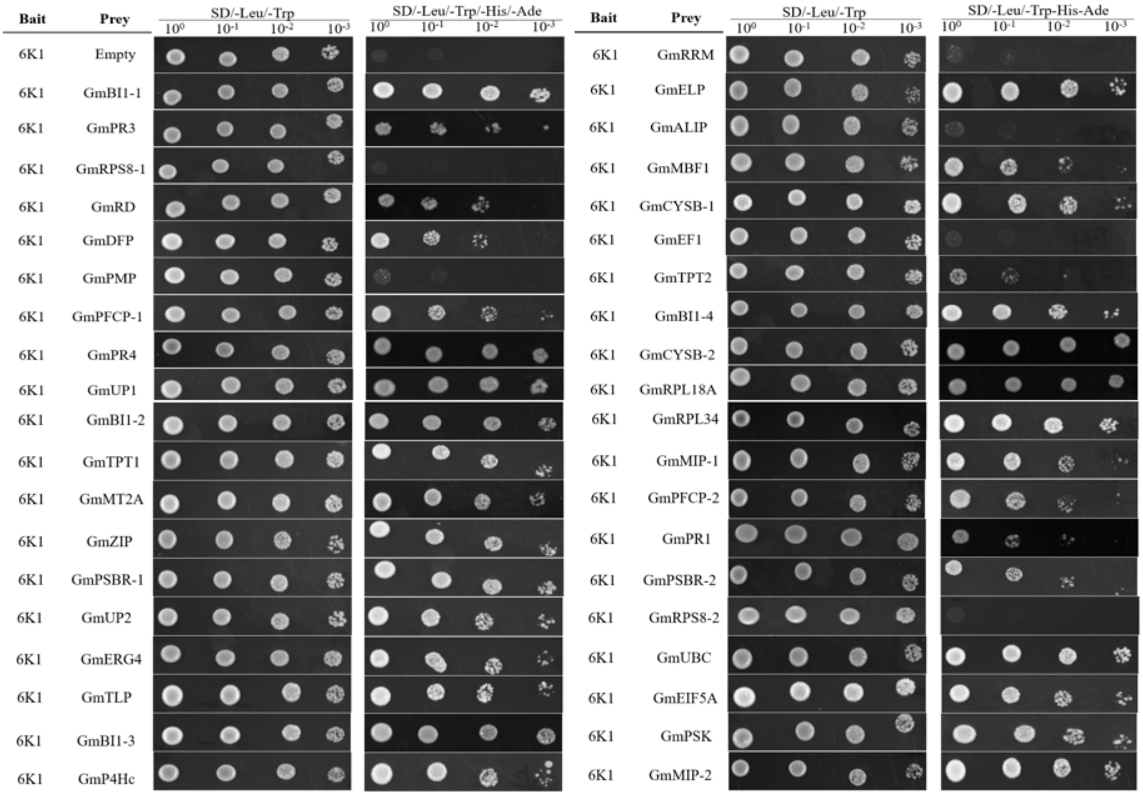

Figure S1. Verification of soybean proteins interacting with 6K1 by yeast two-hybrid (Y2H) assay. The coding sequences of 39 soybean proteins were individually merged into the prey vector pPR3-N and co-transformed with the bait vector pBT3-STE-6K1 into the yeast cells. Yeast cells co-transformed with pBT3-STE-6K1 and pPR3-N were used as the negative control. The yeast grown cells were diluted by 1-, 10-, 100-, and 1000-fold. Each Y2H experiment was repeated at least three times.

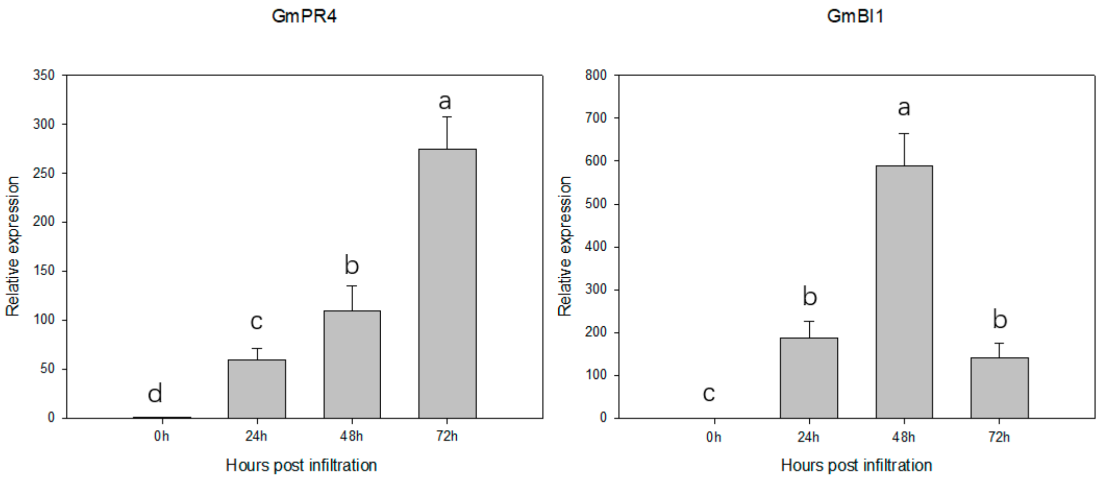

Figure S2. Analysis of the expression of *GmPR4* and *GmBI1* in *N. benthamiana*. The expression levels of *PR4* and *BI1* were examined by qRT-PCR at 0, 24, 48, and 72 hours post infiltration in *N. benthamiana.* Each experiment was repeated three times with error bars indicating standard deviation (SD).The significant differences were tested by one-way ANOVA, $p < 0.05$. Bars with identical letters are not significantly different. The *β-Tubulin* gene (accession No. U91564) of tobacco was used as the internal control to normalize the cDNA.

Table S1. Preliminary screening of soybean proteins interacting with SMV-6K1.

**Protein Annotation**	**Gene**	**Function classification**	**Homologous gene in AT** [1]
Inhibitor of apoptosis-promoting Bax1	*Glyma.01g205200*	Defense related	AT5G47120
Unknown	*Glyma.01G205800*	Unknown	AT2G35658
Chitin recognition protein, PR3	*Glyma.02G042500*	Defense related	AT3G12500
Predicted small membrane protein	*Glyma.02G268700*	Membrane related	AT2G04900
40S ribosomal protein S8	*Glyma.03G086400*	DNA Binding	AT5G59240
Rubredoxin	*Glyma.03G245100*	Metabolism related	AT1G54500
Chitinase-related, PR4	*Glyma.03g247500*	Defense related	AT3G04720
Dehydrin family protein	*Glyma.04g009400*	Defense related	AT1G20440
Predicted membrane protein	*Glyma.04G167300*	Membrane related	AT1G18720
Transporters	*Glyma.04g256300*	Transport related	AT1G22540
Papain family cysteine protease	*Glyma.04g027600*	Defense related	AT4G39090
Triose-phosphate Transporter family	*Glyma.04g067700*	Transport related	AT5G25400
	Glyma.04g111000	Unknown	AT4G10080
Keratinocyte-associated protein 2	*Glyma.04g246100*	Defense related	AT1G77350
Cytochrome b5	*Glyma.04g231800*	Metabolism related	AT5G53560
Protein processing in endoplasmic reticulum	*Glyma.04g121000*	Membrane related	AT4G29330
Inhibitor of apoptosis-promoting Bax1	*Glyma.05g064700*	Defense related	AT5G47120
Translation initiation factor 5A (eIF-5A)	*Glyma.05g024200*	Defense related	AT1G13950
Expansin C-terminal domain	*Glyma.05g065700*	Defense related	AT4G17030
Rubredoxin	*Glyma.05g199700*	Metabolism related	AT5G51010
GTPase Rab5/YPT51 and related small G protein superfamily GTPases	*Glyma.05g231400*	DNA Binding	AT5G45130
Lipase/lipooxygenase, PLAT/LH2 family protein	*Glyma.05g155400*	Metabolism related	AT1G55280
40S ribosomal protein S13	*Glyma.05g052300*	DNA Binding	AT4G00100
40S ribosomal protein S13	*Glyma.05g052600*	DNA Binding	AT4G00100
Predicted membrane protein	*Glyma.06g195300*	Membrane related	AT1G18720

**Protein Annotation**	**Gene**	**Function classification**	**Homologous gene in AT** [1]
Ubiquitin system	*Glyma.06g075000*	Metabolism related	AT4G31160
Protein processing in endoplasmic reticulum	*Glyma.06g317800*	Membrane related	AT4G29330
Organic solute transporter Ostalpha	*Glyma.06g000800*	Transport related	AT4G38360
Triose-phosphate Transporter family	*Glyma.07g201300*	Transport related	AT5G46110
	Glyma.07g035800	Unknown	AT3G13950
	Glyma.07g035700	Unknown	AT3G13950
	Glyma.07g085900	Unknown	AT5G19540
Metallothionein	*Glyma.07g132000*	Defense related	AT3G09390
	Glyma.08g235000	Unknown	AT1G79510
Fe2+/Zn2+ regulated transporter	*Glyma.08g328000*	Transport related	AT1G55910
	Glyma.08g153600	Unknown	No
hotosystem II 10 kDa polypeptide PsbR	*Glyma.08g173700*	Metabolism related	AT1G79040
Synaptobrevin/VAMP-like protein	*Glyma.08g355600*	Transport related	AT1G04760
Molybdate transporter of MFS superfamily	*Glyma.08g208700*	Transport related	AT1G80310
Arabinogalactan peptide	*Glyma.08g021100*	Defense related	AT2G46330
photosystem II reaction center	*Glyma.08g363500*	Metabolism related	ATCG00300
Plant thionin family protein	*Glyma.08g183000*	Defense related	AT1G25275
Cysteine-rich TM module stress tolerance	*Glyma.08g172300*	Defense related	AT2G32190
Ubiquitin-conjugating enzyme	*Glyma.08g042500*	Metabolism related	AT1G70660
Ergosterol biosynthesis ERG4/ERG24 family	*Glyma.08g224200*	Defense related	AT1G50430
Peptidase M50B-like	*Glyma.08g366100*	Metabolism related	AT1G67060
Flavonoid biosynthesis	*Glyma.08g220200*	Defense related	AT5G48930
Complement Clr-like EGF-like	*Glyma.09g274300*	Defense related	AT3G52850
Cytochrome P450	*Glyma.09g282900*	Metabolism related	AT2G29090
Synaptobrevin/VAMP-like protein	*Glyma.09g019800*	Transport related	AT1G04760
Protein chaperone-like protein of por1-like	*Glyma.10g283100*	Metabolism related	AT2G20920
Thaumatin family	*Glyma.10g061800*	Defense related	AT1G20030
Thaumatin family	*Glyma.10g060800*	Defense related	AT1G20030
Thaumatin family	*Glyma.10g061000*	Defense related	AT1G20030

**Protein Annotation**	**Gene**	**Function classification**	**Homologous gene in AT** [1]
40S ribosomal protein S29	*Glyma.10g258500*	DNA Binding	AT3G44010
Glycosyl hydrolases family 17	*Glyma.11g095100*	Metabolism related	AT4G16260
Inhibitor of apoptosis-promoting Bax1	*Glyma.11g037700*	Defense related	AT5G47120
Glycosyl hydrolases family 17	*Glyma.11g095200*	Metabolism related	AT4G16260
Voltage-dependent l-type calcium channel subunit	*Glyma.11g213800*	Transport related	AT5G16550
	Glyma.11g056100	Unknown	AT4G33985
2OG-Fe(II) oxygenase superfamily	*Glyma.11g080700*	Metabolism related	AT5G66060
Amino acid transporter protein	*Glyma.11g234600*	Transport related	AT3G56200
nucleic acid binding	*Glyma.11g117600*	DNA Binding	AT2G21660
Extensin-like protein repeat	*Glyma.11g154900*	Defense related	No
Plant specific mitochondrial import receptor subunit TOM20	*Glyma.11g250100*	Metabolism related	AT1G27390
Uncharacterized membrane protein, predicted efflux pump	*Glyma.12g194000*	Membrane related	AT3G21690
Aluminium induced protein	*Glyma.12g150500*	Defense related	AT4G27450
Multiprotein bridging factor 1	*Glyma.12g129100*	DNA Binding	AT3G58680
Predicted membrane protein	*Glyma.12g093300*	Membrane related	AT2G27290
TLC ATP/ADP transporter	*Glyma.13g362300*	Transport related	AT1G80300
cysteine proteinase inhibitor	*Glyma.13g071800*	Defense related	AT3G12490
elongation factor 1-beta	*Glyma.13g073200*	DNA Binding	AT5G12110
Triose-phosphate Transporter family	*Glyma.13g175100*	Transport related	AT5G46110
RNA BINDING PROTEIN	*Glyma.13g112100*	DNA Binding	AT1G17370
proton pump interactor 1	*Glyma.13g276500*	Transport related	AT4G27500
60S Ribosomal protein L18A	*Glyma.13g261500*	DNA Binding	AT1G17080
Plant PDR ABC transporter associated	*Glyma.13g361900*	Transport related	AT1G15520
Selenoprotein SelK_SelG	*Glyma.13g082200*	Defense related	AT4G08230
Ribulose bisphosphate carboxylase, small chain	*Glyma.13g046200*	Metabolism related	AT5G38410

**Protein Annotation**	**Gene**	**Function classification**	**Homologous gene in AT** [1]
Inhibitor of apoptosis-promoting Bax1	*Glyma.13g211300*	Defense related	AT4G15470
Selenoprotein SelK_SelG	*Glyma.13g082200*	Defense related	AT4G08230
Cystatin domain	*Glyma.13g189500*	Defense related	AT3G12490
DNA repair and recombination proteins	*Glyma.13g110100*	DNA Binding	AT2G06040
Ubiquitin-protein ligase	*Glyma.13g277400*	Metabolism related	AT3G15355
60S Ribosomal protein L34	*Glyma.13g209500*	DNA Binding	AT3G06180
Signal peptidase complex, subunit SPC25	*Glyma.14g061900*	Transport related	AT2G39960
Major intrinsic protein	*Glyma.13g325900*	Transport related	AT2G37170
Amino acid transporter protein	*Glyma.14g054800*	Transport related	AT3G56200
Selenoprotein T	*Glyma.14g194500*	Defense related	AT5G58640
integral component of membrane	*Glyma.14g098800*	Membrane related	No
Calmodulin and related proteins (EF-Hand superfamily)	*Glyma.14g156300*	Defense related	AT1G21550
	Glyma.14g131700	Unknown	AT4G31130
cysteine proteinase1	*Glyma.14g085800*	Defense related	AT1G47128
ADP,ATP CARRIER PROTEIN	*Glyma.15g011500*	Transport related	AT1G80300
Cysteine-rich secretory protein family,PR1	*Glyma.15g062400*	Defense related	AT2G14580
Photosystem II 10 kDa polypeptide PsbR	*Glyma.15g253700*	Metabolism related	AT1G79040
Plant thionin family protein	*Glyma.15g049600*	Defense related	AT1G25275
regulation of transcription, DNA-templated	*Glyma.15g219200*	DNA Binding	AT1G79350
Peptidase family M50	*Glyma.16g037100*	DNA Binding	AT5G35220
	Glyma.16g109900	Unknown	No
40S ribosomal protein S8	*Glyma.16g087700*	DNA Binding	AT5G59240
	Glyma.16g076800	Unknown	No
Light regulated protein Lir1	*Glyma.16g049000*	Metabolism related	AT3G26740
Transmembrane protein	*Glyma.16g160100*	Membrane related	AT1G71780
Strictosidine synthase	*Glyma.16g119000*	Metabolism related	AT3G51430
Ubiquitin-conjugating enzyme	*Glyma.17g032800*	Metabolism related	AT3G17000
ZCF37	*Glyma.17g234800*	Unknown	AT1G59590

Protein Annotation	Gene	Function classification	Homologous gene in AT [1]
Inhibitor of apoptosis-promoting Bax1	*Glyma.17g146500*	Defense related	AT5G47120
RNA recognition motif. (a.k.a. RRM, RBD, or RNP domain)	*Glyma.17g047600*	DNA Binding	AT1G17370
Translation initiation factor 5A (eIF-5A)	*Glyma.17g103100*	DNA Binding	AT1G13950
Amino acid transporters	*Glyma.17g192000*	Transport related	AT5G49630
Cysteine proteinase Cathepsin F	*Glyma.17g254900*	Defense related	AT4G39090
Oligopeptide transporter-related	*Glyma.17g115300*	Transport related	AT2G26690
Phytosulfokine precursor protein	*Glyma.17g233500*	Defense related	AT3G49780
Voltage-dependent l-type calcium channel subunit	*Glyma.18g042900*	Transport related	AT5G16550
Permease family	*Glyma.18g212500*	Transport related	AT3G10960
Peptidase family M50	*Glyma.19g116000*	DNA Binding	AT5G35220
ATPase family associated with various cellular activities (AAA)	*Glyma.19g208300*	DNA Binding	AT1G03000
Transmembrane amino acid transporter protein	*Glyma.19g083900*	Transport related	AT5G40780
Amino acid transporters	*Glyma.19g116400*	Transport related	AT1G58030
Ribulose bisphosphate carboxylase, small chain	*Glyma.19g046600*	Metabolism related	AT5G38410
Aquaporin (major intrinsic protein family)	*Glyma.19G181300*	Transport related	AT3G53420
Molecular chaperone (DnaJ superfamily)	*Glyma.19g112100*	DNA Binding	AT5G18140
Heat shock protein Dnaj domain	*Glyma.20g106600*	Defense related	AT2G20920
HR-like lesion-inducing	*Glyma.20G088200*	Defense related	AT5G43460
	Glyma.U030900	Unknown	No
Peptidase of plants and bacteria	*Glyma.20g129900*	DNA Binding	AT2G15220

[1] AT means *arabidopsis thaliana*.

Table S2. Primers used in this study.

Primer name	Sequences （5′→3′）
sfi-6K1-F	ATTAACAAGGCCATTACGGCCGCCAAAACAGCAACTCAATT
sfi-6K1-R	AACTGATTGGCCGAGGCGGCCCCCTGCACTTTAACATCCTCAC
pPR3-N-F	GTCGAAAATTCAAGACAAGG
pPR3-N-R	AAGCGTGACATAACTAATTAC
Gateway-6K1-F	GGGGACAAGTTTGTACAAAAAAGCAGGCTTCATGGCCAAAACAGCAACTCAATT
Gateway-6K1-R	GGGGACCACTTTGTACAAGAAAGCTGGGTCCTGCACTTTAACATCCTCAC

Primer name	Sequences (5′→3′)
Gateway-BI1-1-F	GGGGACAAGTTTGTACAAAAAAGCAGGCTTCATGGACTCCTTC AATTCCTTCTT
Gateway-BI1-1-R	GGGGACCACTTTGTACAAGAAAGCTGGGTCATCTCTCCTCTTCT TTTTCTTCTCA
Gateway-PR3-F	GGGGACAAGTTTGTACAAAAAAGCAGGCTTCATGAAAAACAT GAAATTGTGTTCGG
Gateway-PR3-R	GGGGACCACTTTGTACAAGAAAGCTGGGTCAGCAAATGGCCTT TGATTGTT
Gateway-RD-F	GGGGACAAGTTTGTACAAAAAAGCAGGCTTCATGGGCATATA CTATGGTTGTCCT
Gateway-RD-R	GGGGACCACTTTGTACAAGAAAGCTGGGTCTTGGAGGAAGTA GCCACTCA
Gateway-PR4-F	GGGGACAAGTTTGTACAAAAAAGCAGGCTTCATGGCAAAGGT ATCTCTGTTTGT
Gateway-PR4-R	GGGGACCACTTTGTACAAGAAAGCTGGGTCGTCACCGCAGTCG ACGAA
Gateway-DFP-F	GGGGACAAGTTTGTACAAAAAAGCAGGCTTCATGGCAGACGA GACCCAGA
Gateway-DFP-R	GGGGACCACTTTGTACAAGAAAGCTGGGTCACTTGTTCTGGGT CTCGTCTG
Gateway-PMP-F	GGGGACAAGTTTGTACAAAAAAGCAGGCTTCATGGGTCTGTTG GATCTGGAG
Gateway-PMP-R	GGGGACCACTTTGTACAAGAAAGCTGGGTCAGAATGTTTCTTT TGTTTCTTGGC
Gateway-PFCP-1-F	GGGGACAAGTTTGTACAAAAAAGCAGGCTTCATGGCTAATCTC TCAATCTTGTTCT
Gateway-PFCP-1-R	GGGGACCACTTTGTACAAGAAAGCTGGGTCATGGCTAATCTCT CAATCTTGTTCT
Gateway-UP1-F	GGGGACAAGTTTGTACAAAAAAGCAGGCTTCATGGAATCTAAT CCAACAACCAT
Gateway-UP1-R	GGGGACCACTTTGTACAAGAAAGCTGGGTCTCTCATACTCATC ACAGGCCTCA
Gateway-BI1-2-F	GGGGACAAGTTTGTACAAAAAAGCAGGCTTCATGGACACCTTC TTCAATTCTCA
Gateway-BI1-2-R	GGGGACCACTTTGTACAAGAAAGCTGGGTCATCTCTCCTTTTCT TCTTCTTCTCA
Gateway-TPT1-F	GGGGACAAGTTTGTACAAAAAAGCAGGCTTCATGGAGTCGCG AGTGCTG
Gateway-TPT1-R	GGGGACCACTTTGTACAAGAAAGCTGGGTCTGCTGCTTTCGCTT GTCG
Gateway-MT2A-F	GGGGACAAGTTTGTACAAAAAAGCAGGCTTCATGTCTTGCTGC GGTGGTA
Gateway-MT2A-R	GGGGACCACTTTGTACAAGAAAGCTGGGTCCTTGCAAGTGCAG GGGTTGCA
Gateway-ZIP-F	GGGGACAAGTTTGTACAAAAAAGCAGGCTTCATGTCACCTTCT TTTTGCACATC
Gateway-ZIP-R	GGGGACCACTTTGTACAAGAAAGCTGGGTCGGTGTCCCAAATC ATTACCAC
Gateway-PSBR-1-F	GGGGACAAGTTTGTACAAAAAAGCAGGCTTCATGGCCTCTTCA GTGATGGC
Gateway-PSBR-1-R	GGGGACCACTTTGTACAAGAAAGCTGGGTCTTGCGCCAAAGC ACTTGTGT

Primer name	Sequences （5′→3′）
Gateway-UP2-F	GGGGACAAGTTTGTACAAAAAAGCAGGCTTCATGAAGAAGAT GGTGGCTGC
Gateway-UP2-R	GGGGACCACTTTGTACAAGAAAGCTGGGTCTAGCATTATAAGT TTGAAGAATAAA
Gateway-ERG4-F	GGGGACAAGTTTGTACAAAAAAGCAGGCTTCATGGGGGCCAC CGTT
Gateway-ERG4-R	GGGGACCACTTTGTACAAGAAAGCTGGGTCGTATATTCCTGGA ATGATTCTGTAA
Gateway-TLP-F	GGGGACAAGTTTGTACAAAAAAGCAGGCTTCATGACGACCAC CCGTGTTG
Gateway-TLP-R	GGGGACCACTTTGTACAAGAAAGCTGGGTCAGGGCAGAATGT GATGGCATA
Gateway-BI1-3-F	GGGGACAAGTTTGTACAAAAAAGCAGGCTTCATGGACGCCTTC AATTCCT
Gateway-BI1-3-R	GGGGACCACTTTGTACAAGAAAGCTGGGTCATCTCTTCTCTTCT TTTTCTTCTCA
Gateway-P4Hc-F	GGGGACAAGTTTGTACAAAAAAGCAGGCTTCATGGCGAAACC AAGGTACTCTC
Gateway-P4Hc-R	GGGGACCACTTTGTACAAGAAAGCTGGGTCAGCTTTGTATTCG CTGACACGCATC
Gateway-RRM-F	GGGGACAAGTTTGTACAAAAAAGCAGGCTTCATGGCTTCTGCA GATGTTGAGT
Gateway-RRM-R	GGGGACCACTTTGTACAAGAAAGCTGGGTCATTTCTCCAGTTTC CATCGGATC
Gateway-ELP-F	GGGGACAAGTTTGTACAAAAAAGCAGGCTTCATGGCTTCCTTT GTATCCTTCCT
Gateway-ELP-R	GGGGACCACTTTGTACAAGAAAGCTGGGTCGGCATGGGTGTTG TCCTCTA
Gateway-ALIP-F	GGGGACAAGTTTGTACAAAAAAGCAGGCTTCATGTTGTCCATT TTTCACAAGGGGTT
Gateway-ALIP-R	GGGGACCACTTTGTACAAGAAAGCTGGGTCATGCTGGGTCCAT TCCATCCAATTA
Gateway-MBF1-F	GGGGACAAGTTTGTACAAAAAAGCAGGCTTCATGTCAGGTGTT GGCCCTCTATCTC
Gateway-MBF1-R	GGGGACCACTTTGTACAAGAAAGCTGGGTCTTTCTTGCCACGC AATTTTGCTC
Gateway-RPS8-F	GGGGACAAGTTTGTACAAAAAAGCAGGCTTCATGGGTATCTCC AGAGATTCTATGC
Gateway-RPS8-R	GGGGACCACTTTGTACAAGAAAGCTGGGTCAGCAGCACCCTTT CCCTTCTT
Gateway-CYSB-1-F	GGGGACAAGTTTGTACAAAAAAGCAGGCTTCATGGCAGCACTT GGTGGCAA
Gateway-CYSB-1-R	GGGGACCACTTTGTACAAGAAAGCTGGGTCTGCAGGTGCATCT CCAACAAGCT
Gateway-EF1-F	GGGGACAAGTTTGTACAAAAAAGCAGGCTTCATGGCCGTTACC TTCTCAAATCTC
Gateway-EF1-R	GGGGACCACTTTGTACAAGAAAGCTGGGTCGATTTTGTTGAAT GCAACAATGTCACAGC
Gateway-TPT2-F	GGGGACAAGTTTGTACAAAAAAGCAGGCTTCATGGAGTCGCG AGTGCTG
Gateway-TPT2-R	GGGGACCACTTTGTACAAGAAAGCTGGGTCTGCTGCTTTTGCTT GTCGC

Gateway-BI1-4-F	GGGGACAAGTTTGTACAAAAAAGCAGGCTTCATGTTTGAGCCACAACAGCTATA
Gateway-BI1-4-R	GGGGACCACTTTGTACAAGAAAGCTGGGTCATTATTTGCCTCCCTGAGGAT
Gateway-CYSB-2-F	GGGGACAAGTTTGTACAAAAAAGCAGGCTTCATGAGAGCATCAAACTCTTCTTCTT
Gateway-CYSB-2-R	GGGGACCACTTTGTACAAGAAAGCTGGGTCGGAATGATCTTGTTCCATCTGATT
Gateway-RPL18A-F	GGGGACAAGTTTGTACAAAAAAGCAGGCTTCATGACCGACGAAGACAAACCC
Gateway-RPL18A-R	GGGGACCACTTTGTACAAGAAAGCTGGGTCCCAATCATCTACTCCCTTTGTCAC
Gateway-RPL34-F	GGGGACAAGTTTGTACAAAAAAGCAGGCTTCATGATTCCCAAGATTTCTCCGA
Gateway-RPL34-R	GGGGACCACTTTGTACAAGAAAGCTGGGTCAAAGGATGTCAACCTCCCAAGA
Gateway-MIP-1-F	GGGGACAAGTTTGTACAAAAAAGCAGGCTTCATGGCGAAAGACGTTGAGC
Gateway-MIP-1-R	GGGGACCACTTTGTACAAGAAAGCTGGGTCAGCGTTGCTCCTGAAGGAT
Gateway-PFCP-2-F	GGGGACAAGTTTGTACAAAAAAGCAGGCTTCATGTTCACTGCACCTTTTCCC
Gateway-PFCP-2-R	GGGGACCACTTTGTACAAGAAAGCTGGGTCAGCACTGCTGACCTTGTTCTTAC
Gateway-PR1-F	GGGGACAAGTTTGTACAAAAAAGCAGGCTTCATGGGGTTGTGCAAGGTTTC
Gateway-PR1-R	GGGGACCACTTTGTACAAGAAAGCTGGGTCGTAGGGTCTTTGGCC
Gateway-PSBR-2-F	GGGGACAAGTTTGTACAAAAAAGCAGGCTTCATGGCCTCTTCAGTGATGGC
Gateway-PSBR-2-R	GGGGACCACTTTGTACAAGAAAGCTGGGTCTTGAACCAAAGCACTTGTGTTGTAG
Gateway-UBC-F	GGGGACAAGTTTGTACAAAAAAGCAGGCTTCATGGCGGAGAAACACAACCT
Gateway-UBC-R	GGGGACCACTTTGTACAAGAAAGCTGGGTCAGATTCATCCATGAAAACAGCACC
Gateway-EIF5A-F	GGGGACAAGTTTGTACAAAAAAGCAGGCTTCATGTCGGACGAAGAGCACC
Gateway-EIF5A-R	GGGGACCACTTTGTACAAGAAAGCTGGGTCGTTCTTTGGCCCAATATCCTTGA
Gateway-MIP-2-F	GGGGACAAGTTTGTACAAAAAAGCAGGCTTCATGGCCAAAGACCTCGAAAC
Gateway-MIP-2-R	GGGGACCACTTTGTACAAGAAAGCTGGGTCCAGGTTTGAGGAGCTCCTGAAA
Gateway-PSK-F	GGGGACAAGTTTGTACAAAAAAGCAGGCTTCATGTCTAAAGTGGCCACCCTC
Gateway-PSK-R	GGGGACCACTTTGTACAAGAAAGCTGGGTCAGGTTTGGGTTTATGCTTCTGA
qRT-Tubulin-F	GGAGTTCACAGAGGCAGAG
qRT-Tubulin-R	CACTTACGCATCACATAGCA
qRT-BI1-F	TCTTCTTCGAGAAGCCGCTG
qRT-BI1-R	GCCAACGTGGTGAGAAAACC

Primer name	Sequences （5′ →3′ ）
qRT-PR4-F	GCCTCTGCTCAGAGTGCTAC
qRT-PR4-R	GGTCCACAGAAAGCTGTCCA
pGD-PR4-F	CGGATCATCTAGAACTAGTGGATCCATGGCAAAGGTATCTCTG TTTGTG
pGD-PR4-R	TGATATCGAATTCCTGCAGCCCGGGGTCACCGCAGTCGACGAA
pGD-BI1-F	CGGATCATCTAGAACTAGTGGATCCATGGACACCTTCTTCAATT CTCA
pGD-BI1-R	TGATATCGAATTCCTGCAGCCCGGGATCTCTCCTTTTCTTCTTCT TCTCA
qRT-β-Tubulin-F	GGATGCCACTGCTGATGAC
qRT-β-Tubulin-R	AGTTGCCTTCATGATCGTACTCT
